# Urachal Mucinous Cystic Tumor of Low Malignant Potential with Concurrent Sigmoid Colon Adenocarcinoma

**DOI:** 10.1155/2019/1434838

**Published:** 2019-06-25

**Authors:** Kelly Brennan, Paul Johnson, Heather Curtis, Thomas Arnason

**Affiliations:** ^1^Faculty of Medicine, Dalhousie University, Halifax, Nova Scotia, Canada; ^2^Department of General Surgery, Dalhousie University, Halifax, Nova Scotia, Canada; ^3^Department of Radiology, Dalhousie University, Halifax, Nova Scotia, Canada; ^4^Department of Pathology, Dalhousie University, Halifax, Nova Scotia, Canada

## Abstract

Urachal mucinous tumors are rare neoplasms with behaviour that can range from relatively benign to malignancy that can spread distantly or throughout the peritoneum as pseudomyxoma peritonei or peritoneal carcinomatosis. Here we describe a unique case of urachal mucinous cystic tumor of low malignant potential confined to an intact cyst at the dome of the urinary bladder, without rupture or peritoneal spread. The urachal mucinous tumor was an incidental finding on a staging CT scan performed for sigmoid colon adenocarcinoma. We believe that this case illustrates a potential diagnostic pitfall which could have prognostic and therapeutic implications. Due to the intestinal phenotype of these neoplasms, a urachal tumor of low malignant potential could be mistaken for metastatic spread from a colonic adenocarcinoma in the rare situation such as this case, where the two neoplasms occur concurrently.

## 1. Introduction

Urachal neoplasms are thought to arise from neoplastic transformation of remnant urachal tissue left from incomplete regression of the urachus in fetal development [1–11]. Most urachal neoplasms are epithelial (glandular) neoplasms (see classification in Table 1), typically with an intestinal phenotype [1–11]. The spectrum of cystic urachal mucinous neoplasms (described in Table 2), including mucinous cystadenoma, mucinous cystic tumor of low malignant potential, and mucinous cystadenocarcinoma [12], is similar to the morphologic spectrum of appendiceal [13] and ovarian [12, 14] intestinal-type mucinous neoplasms. Consequently, the absence of a known primary glandular neoplasm at another anatomical site has been put forward as a criterion for pathologic diagnosis of a urachal mucinous neoplasm [12, 15]. However, in this report we describe a unique patient with a clinical presentation that defies this convention. This patient presented with a urachal mucinous cystic tumor of low malignant potential and a concurrent invasive adenocarcinoma of the sigmoid colon. We believe that the differences in morphology, beta-catenin immunohistochemistry, and the distinct anatomical locations of the two tumors rule out metastasis from one site to the other.

## 2. Methods

Care was provided at a tertiary care teaching hospital and the patient provided written consent for a review of medical records and for publication of a case report, in accordance with institutional policy. Data regarding clinical history, diagnostic imaging, and pathology were collected retrospectively.

## 3. Results

### 3.1. Case Presentation

**Figure 1:**
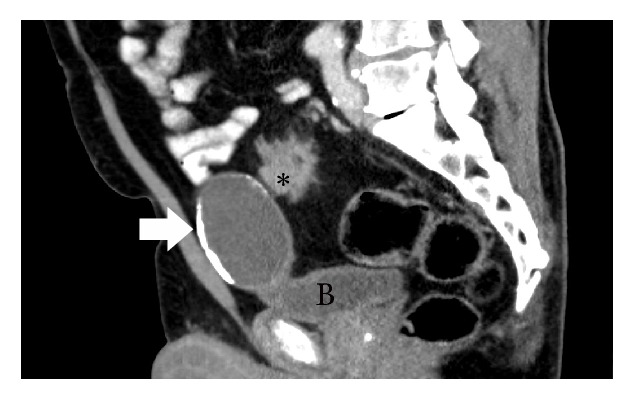
Sagittal image from contrast enhanced CT demonstrating a 6.9 cm rim calcified cyst (see Arrow) arising from the dome of the urinary bladder (labelled “B”) corresponding to a urachal cystic tumor of low malignant potential. Immediately posterior to this is the 6.5 cm sigmoid colon adenocarcinoma (labelled *∗*), represented as circumferentially thickened bowel with luminal narrowing and irregular serosal surface seen in cross-section.

The patient, a 67-year-old male, underwent a colonoscopy after a positive Fecal Immunochemical Test result in the province's colon cancer screening program. On review of systems, the patient reported a change in bowel habits, specifically cramping and a sense of urgency. His past medical history was unremarkable apart from hypertension and hyperlipidemia. Colonoscopy revealed a stricturing malignancy in the distal sigmoid colon. Biopsies were diagnostic of colonic adenocarcinoma. A CT scan of the chest, abdomen, and pelvis demonstrated a 6.5 cm segment of circumferential wall thickening in the sigmoid colon, 20 cm from the anal verge. The CT scan also identified an incidental, 6.9 x 4.8 cm rim calcified cystic lesion arising from the dome of the urinary bladder, suspected to represent a bladder diverticulum or a urachal cyst (CT scan illustrated in Figure 1). At the time of surgery, there was no evidence of pseudomyxoma peritonei or peritoneal carcinomatosis. The sigmoid colon cancer and the cystic lesion at the dome of the bladder were separate entities and were not physically connected. A sigmoid resection with primary anastomosis was performed. The cystic lesion at the dome of the bladder was resected separately during the same procedure and sent as a second specimen to pathology.

### 3.2. Pathologic Findings

**Figure 2:**
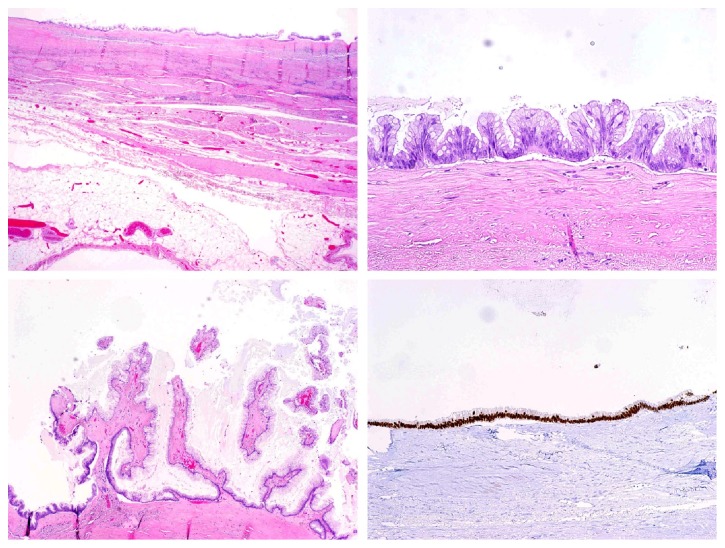
(a) Cyst wall showing fibromuscular wall and surface epithelial lining (20X Magnification). (b) Cyst epithelial lining with nuclear pseudostratification (H&E 200X Magnification). (c) Cyst with an area of simple papillary architecture (H&E 100X Magnification). (d) Cyst showing epithelial expression of CDX2 by immunohistochemistry (100X Magnification).

On gross examination, the cyst from the dome of the bladder measured 9.0 x 5.5 x 5.0 cm. It was unilocular and thin walled (0.1-0.6 cm thick), partially calcified, and lacked any grossly identifiable papillary projections or solid component. The cyst content was mucin. On H&E microscopy, the epithelial lining consisted of a single layer of cuboidal to columnar epithelial cells with an intestinal phenotype, including scattered goblet cells (illustrated in Figure 2). The nuclei of the cyst epithelial lining cells were elongated and hyperchromatic (pencillate) throughout, in the pattern of intestinal type low-grade dysplasia. There were areas of villous and simple papillary architecture, reminiscent of a low grade appendiceal mucinous neoplasm (LAMN) or an ovarian mucinous borderline tumor. Immunohistochemical stains showed that the epithelial lining of the cyst was positive for CK20 and CDX2, while negative for CK7 (intestinal immunophenotype). Beta-catenin immunohistochemistry showed membranous expression in the epithelial lining, with complete absence of nuclear expression. The lumen of the cyst contained acellular mucin, which dissected in some areas into the partially calcified cyst wall, but did not reach the serosal surface. There was smooth muscle in part of the cyst wall, but in most areas, the cyst wall was collagenous without muscle. The cyst was felt to be best classified as a urachal mucinous cystic tumor of low malignant potential, based on the classification system described by Paner et al. [12].

**Figure 3:**
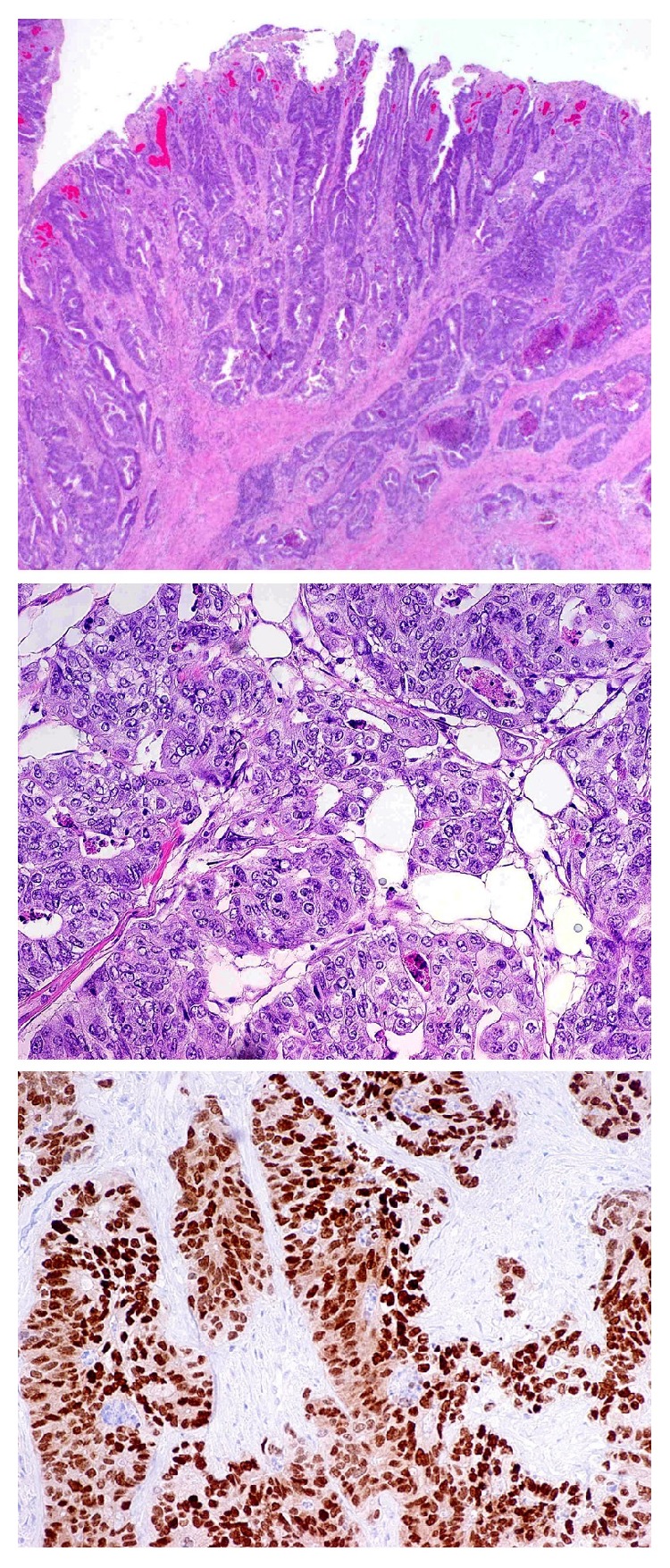
(a) Invasive colonic adenocarcinoma (20X Magnification). (b) Invasive colonic adenocarcinoma (200X Magnification). (c) Colonic adenocarcinoma showing epithelial expression of CDX2 (200X Magnification).

The sigmoid colon contained a 5.5 cm circumferential mass. Histologically, the tumor was a moderately differentiated invasive adenocarcinoma, not otherwise specified (illustrated in Figure 3). Notably, there was no mucinous component in the colon adenocarcinoma. By immunohistochemistry, the adenocarcinoma was positive for CK20, CDX2 and negative for CK7. Beta-catenin immunohistochemistry was positive with nuclear localization in tumor cells and weaker membranocytoplasmic expression. The stage was pT4aN0 (AJCC 8th edition TNM stage), with 17 negative lymph nodes and negative margins. Although the tumor reached the serosal surface, there was no evidence of invasion of other structures, including the cyst.

### 3.3. Follow-Up

There were no postoperative complications. The patient did not receive systemic chemotherapy or radiation therapy following surgery. Nine months after surgery, he presented to the emergency department with a productive cough and a chest X-ray identified two left upper lobe lung nodules, 7mm and 11mm in diameter, suspicious for metastases. The two lung lesions were removed by video assisted thoracoscopic surgery. Histologically, the lung lesions were invasive adenocarcinoma with no mucinous component. The morphology was identical to the sigmoid colon adenocarcinoma. Six months after resection of the lung metastases (18 months after presentation), the patient had no further evidence of metastasis or local recurrence.

## 4. Discussion

The urachus is a vestigial remnant derived from the embryonic tissue connecting the allantois to the urinary bladder [34]. In fetal development, the urachus regresses to form the median umbilical ligament [35]. Incomplete regression of the urachus can give rise to urachal fistulas, cysts, and rarely neoplasms later in life [34]. Urachal neoplasms account for less than 0.5% of neoplasms of the urinary bladder [15]. Most urachal neoplasms have a glandular phenotype [3]. There is some variation in the nomenclature used in the literature to describe urachal neoplasms [10, 12], especially the mucinous cystic neoplasms like the one described here [10, 16–19, 24, 26, 27, 29–33]. Amin et al. and Paner et al. have put forward classification systems to improve consistency in naming both the epithelial neoplasms of the urachus in general and more specifically the mucinous cystic neoplasms (Table 1) [10, 12].

Forty-two cases of urachal mucinous cystic neoplasms have been described in the literature, in eighteen case reports and a case series of 24 patients, summarized in Table 2. Only one of the 42 cases was described as having a concurrent neoplasm (a germ cell tumor). No prior mucinous cystic tumor of the urachus has been described in association with a concurrent glandular neoplasm at another site, and some authors suggest that the finding of a concurrent intestinal type glandular neoplasm should exclude the diagnosis of a urachal mucinous neoplasm [12, 15]. However, we think this case report defies that convention. We do not think that concurrent adenocarcinoma should be exclusion criteria in the diagnosis of urachal mucinous cystic neoplasms. While this patient's sigmoid colon adenocarcinoma and urachal neoplasm both have an intestinal phenotype with the same immunohistochemical profile (CK20 positive, CDX2 positive, and CK7 negative), we do not think it is reasonable to conclude that one tumor could represent metastatic spread from one to the other, as the architecture of the two neoplasms is far too distinct. The mucinous cyst is completely lacking the complex (cribriform) and destructive invasion of the sigmoid adenocarcinoma. The adenocarcinoma also lacked mucinous differentiation. Another important difference includes the results of nuclear beta-catenin expression. Specifically, there was an increased expression of beta-catenin by immunohistochemistry, localized to the nuclei of the colorectal adenocarcinoma. This is common in colonic adenocarcinomas and is thought to be mainly attributable to mutations in the adenomatous polyposis coli (APC) gene [20]. In contrast, the urachal mucinous cystic tumor of low malignant potential lacked nuclear beta-catenin expression. Nuclear beta-catenin expression is reportedly rare within the entire spectrum of urachal mucinous neoplasms, and beta-catenin immunohistochemistry has been suggested as a way to distinguish these tumors from metastatic colorectal cancer [21, 22]. Finally, it seems unreasonable to suggest that the colon cancer arose from malignant degeneration of the cyst, when there is no direct connection between the two tumors and no evidence of spread in the peritoneal cavity, as pseudomyxoma peritonei or carcinomatosis.

The most significant potential pitfall in this case would have been a pathologist interpreting the urachal mucinous neoplasm as a cystic metastasis from the colon cancer, perhaps due to a lack of awareness of urachal mucinous neoplasms. The potential risks of such an interpretation could include unnecessary systemic therapy, or a potential second surgical procedure for peritoneal cytoreduction and intraperitoneal chemotherapy (Sugarbaker procedure). This patient has been treated with only one abdominal surgery. He developed lung metastases that were surgically resected. There has been no evidence of local recurrence or peritoneal spread on surveillance imaging. We hope that this case will prove informative to pathologists, surgeons, and oncologists managing a similar scenario in the future, and we hope that this story will support those teams' decisions to manage a case like this as two independent, concurrent neoplasms.

## Figures and Tables

**Table 1:** Classification of epithelial neoplasms of urachal origin with emphasis on the cystic mucinous neoplasms, modified from Paner et al., 2016, & Amin et al., 2014 [10, 12].

*Glandular neoplasms*
(i) Adenoma

(ii) Cystic mucinous neoplasms:

(a) Mucinous cystadenoma (cystic tumor with a single layer of mucinous columnar epithelium, with no atypia)

(b) Mucinous cystic tumor of low malignant potential (cystic tumor with areas of epithelial proliferation, including papillary formation
and low-grade atypia/dysplasia)

(c) Mucinous cystic tumor of low malignant potential with intraepithelial carcinoma (cystic tumor with significant epithelial
stratification and unequivocal malignant cytological features and often with stroma-poor papillae and cribriform pattern)

(d) Mucinous cystadenocarcinoma with microinvasion (stromal invasion <2mm and comprising <5% of the tumor)

(e) Frankly invasive mucinous cystadenocarcinoma (stromal invasion that is more extensive than 2mm and 5%)

(iii) Non-cystic adenocarcinoma

*Non-glandular neoplasms*

(i) Urothelial neoplasm

(ii) Squamous cell neoplasm

(iii) Neuroendocrine neoplasm

(iv) Mixed-type neoplasm

NOS: not otherwise specified.

**Table 2:** Summary of literature review of urachal mucinous tumors.

Primary Study Author	Year	N	Age	Sex	PMP	Size (cm)	Diagnosis	Concurrent neoplasms	Presentation/symptoms	Extent of Surgical Treatment
Agrawal [16]	2014	1	50	M	Yes	8	low grade mucinous urachal neoplasm	No	Abdominal pain	Cystic mass resection, partial cystectomy, extended parietal peritonectomy

Amin [10]	2014	24	24-80 (mean 47)	9 M 14 F 1 UNK	Unk	0.8-13 (mean 5)	4 mucinous cystadenomas, 20 Mucinous cystic tumors of low malignant potential	Not mentioned, 1 case had a concurrent sigmoid colectomy performed	Hematuria, umbilical mass, incidental finding, suprapubic mass, mucusuria, abdominal pain, bladder dome nodule, urgency, obstruction, umbilical discharge, pelvic mass, midline cystic mass	Cystic mass resection, partial cystectomy, umbilectomy

Carr [17]	2001	1	72	M	No	4	Urachal mucinous tumor of uncertain malignant potential	No	Hematuria (microscopic), nocturia	Cystic mass resection, partial cystectomy

Chahal [18]	2015	1	37	M	No	4	Mucinous cystic tumor of low malignant potential (MCTLMP)	Yes - stage pT2, non-stem germ cell tumor	Incidental finding	Partial cystectomy, left hydrocelectomy

Choi [19]	2012	1	29	F	No	5.5	Urachal mucinous tumor of uncertain malignant potential	No	Right flank pain	Cystic mass resection, partial cystectomy

Fahed [20]	2012	1	66	M	No	9	Adenocarcinoma in situ	No	Lower abdominal pain and groin pain	Cystic mass resection, partial cystectomy

Gupta [21]	2014	1	15	F	No	4.5	Low grade mucinous neoplasm with uncertain malignant potential	No	Lower abdominal pain	Cystic mass resection

Hubens [22]	1995	1	40	M	No	8	Urachal adenoma	No	Incidental finding	Cystic mass resection, cholecystectomy

Hull [23]	1994	1	32	M	No	14	Urachal Cystadenoma	No	Incidental finding	Cystic mass resection

Nozaki [24]	2011	1	37	M	Yes	5	Mucinous borderline tumor of low malignant potential	No	Abdominal pain	Cystic mass resection, extensive peritonectomy

Pasternak [25]	2014	1	28	F	No	8	Mucinous urachal neoplasm of low malignant potential	No	Incidental finding	Cystic mass resection, partial cystectomy, umbilectomy, omentectomy, bilateral pelvic lymphadenectomy

Paul [26]	1998	1	68	M	No	3	Stage 0 mucinous adenocarcinoma in situ of the urachus	No	Hematuria, mucusuria	Cystic mass resection, partial cystectomy

Prakash [27]	2014	1	58	M	No	10	Complex mucinous cystadenoma of undetermined malignant potential of the urachus	No	Lower abdominal pain	Cystic mass resection

Saha [28]	2011	1	60	F	No	3	Mucinous cystadenoma	No	Urinary frequency	Cystic mass resection

Schell [29]	2009	1	70	F	No	15.5	Complex mucinous cystadenoma of undetermined malignant potential of the urachus	No	Lower abdominal mass	Cystic mass resection, partial cystectomy

Shinohara [30]	2006	1	54	M	Yes	9	Mucinous cystic tumour with low malignant potential	No	Found incidentally during left inguinal hernia repair	Cystic mass resection, partial cystectomy, intraperitoneal lavage

Stenhouse [31]	2003	1	54	M	Yes	14	Mucinous neoplasm of uncertain malignant potential	No	Abdominal pain, rectal bleeding	Not available

Wang [32]	2016	1	54	M	No	4	Urachal mucinous cystic tumor of low malignant potential	No	Hip pain	Cystic mass resection, partial cystectomy, umbilectomy

Wu [33]	2017	1	41	M	No	3	Urachal cystadenoma with unknown malignant potential	No	Lower abdominal swelling and pain	Cystic mass resection, partial cystectomy

PMP: pseudomyxoma peritonei; UNK: unknown.
